# Honeybee pupal length assessed by CT-scan technique: effects of Varroa infestation, developmental stage and spatial position within the brood comb

**DOI:** 10.1038/s41598-019-46474-4

**Published:** 2019-07-23

**Authors:** Elena Facchini, Laura Nalon, Maria Elena Andreis, Mauro Di Giancamillo, Rita Rizzi, Michele Mortarino

**Affiliations:** Department of Veterinary Medicine, University of Milano, via G. Celoria 10, 20133 Milan, Italy

**Keywords:** X-ray tomography, Entomology

## Abstract

Honeybee pupae morphology can be affected by a number of stressor, but *in vivo* investigation is difficult. A computed tomography (CT) technique was applied to visualize a comb’s inner structure without damaging the brood. The CT scan was performed on a brood comb containing pupae developed from eggs laid by the queen during a time window of 48 hours. From the CT images, the position of each pupa was determined by recording coordinates to a common reference point. Afterwards, every brood cell was inspected in order to assess the developmental stage of the pupa, the presence of *Varroa destructor*, the number and progeny of foundress mites. Using data on 651 pupae, the relationships between varroa infestation status, developmental stage and spatial position of the pupa within the brood comb, and its length were investigated. Pupae at 8 post-capping days were shorter than pupae at 7 post-capping days. Pupae in infected cells were significantly shorter than those in varroa-free cells and this effect was linked both to mite number and stage and to the position in the comb. Overall, the results suggest that the CT-scan may represent a suitable non-invasive tool to investigate the morphology and developing status of honeybee brood.

## Introduction

In recent years, honeybee colony losses have been recorded throughout Europe and the World^1–3^. While a multitude of causative factors for this phenomenon have been extensively debated, now infestation with the invasive ectoparasitic mite *Varroa destructor* is considered one of the most significant causes for colony losses^4^. The mites depend on honey bee brood for reproduction, and the reproductive cycles of host and parasite are tightly linked to each other^5^. Within the isolated and protected environment of a capped cell, the reproducing mites and their offspring feed on the developing honey bee pupae. While the native host *Apis cerana* has evolved a multitude of behavioral adaptations to limit the damage inflicted by the parasite, heavy mite infestation in colonies of *A. mellifera* causes severe damage, typically associated with secondary virus infections and a complex of symptoms known as varroosis, and will eventually lead to colony collapse. At honeybee individual level, it was reported that varroa infestation causes weight loss and reduced life span^6–9^. Moreover, it was reported that multiple infestation of mites in one cell can cause shrinkage of the bee abdomen and increase the risk of developing deformed wings^10^.

The alteration of honeybee pupae morphology including size and length can be considered of value to assess the negative effects of mite infestation of the colony^6–9^. Current methods for varroa load assessment in the brood, as for instance opening a random sample of capped brood cells (n = 200) and measuring the percentage of infested cells, are invasive, partially or totally destructive and time consuming^11^. For research purpose it is important to develop innovative and non-invasive methods to assess the brood mite infestation degree of a colony. Among the currently available imaging diagnostic techniques, computed tomography (CT) imaging technique employs x-rays to produce cross-sectional images (slices) of a scanned object, allowing the visualization of its inner structures without inherent damages to live tissues and materials. In particular, µCT is commonly employed for the 3D visualization of inner structures on a small scale, *i.e*. for morphological investigation of invertebrates^12^. Benchtop µCT systems provide high penetrating power and high resolution images, but scanning typically takes some hours to be completed and suffers for sample size limitations^12^. On the other hand, medical CT devices are optimized for qualitative viewing of larger organisms and objects, providing much lower resolution but also less harmful radiation and reduced scanning time compared to µCT^13^. In this study, medical CT and image analysis approach coupled with brood manual inspection was used to clarify the relationship between *Varroa destructor* infestation status and pupa length, taking into consideration other factors such as the spatial position of the pupa within the comb and its developmental stage. Also, the distribution of infected cells throughout the brood area of the comb was investigated.

## Results

**Figure 1:**
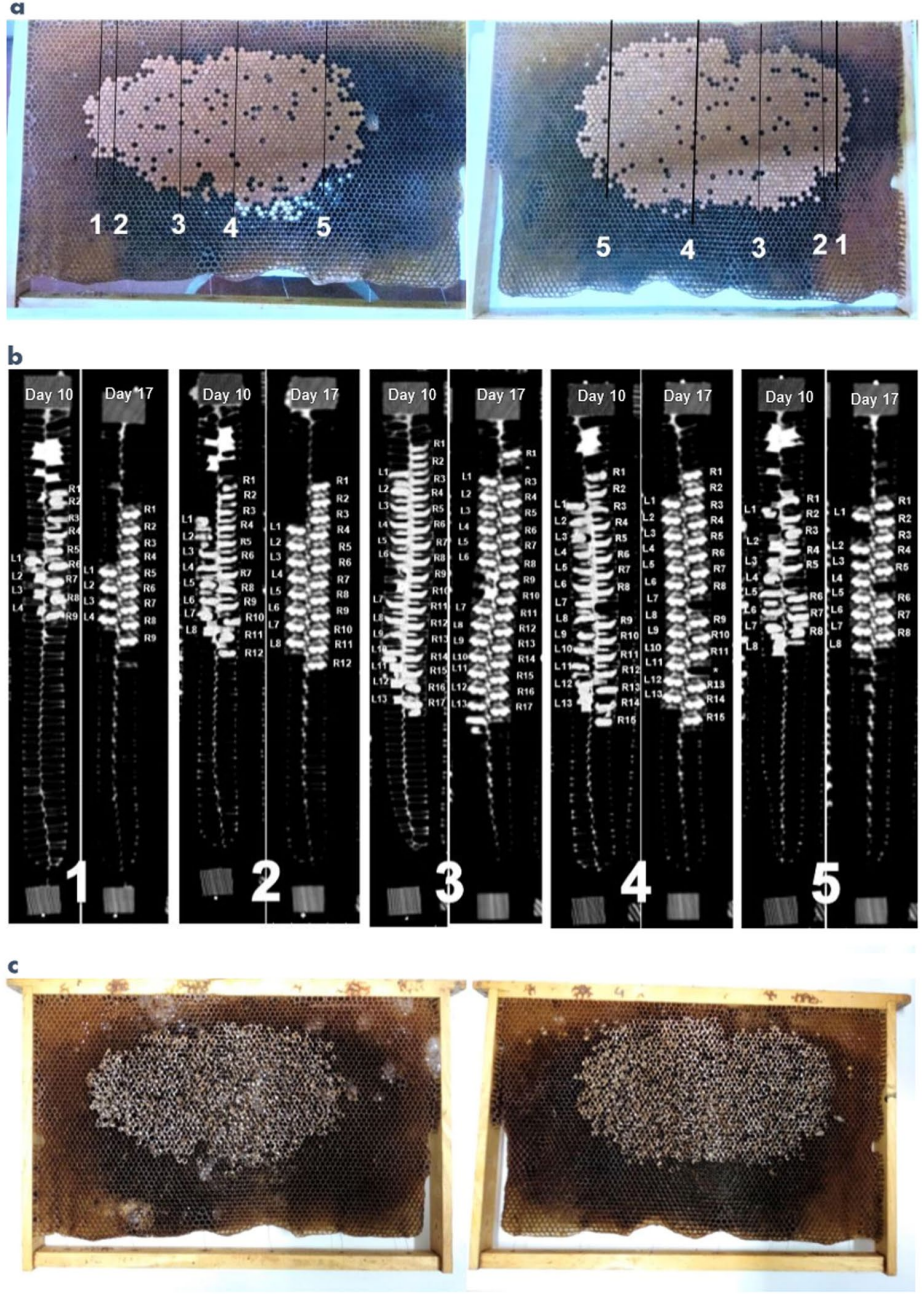
Honeybee brood area investigated by medical CT-scan and manual uncapping. Panel a, frontal picture of both left and right side of the brood comb. Panel b, pupal development across five sections of the brood comb assessed by the two CT scans. The five coupled images of the coronal plane of the comb show the honeybee larvae on Day 10 on the left (Ln) and right (Rn) side of the comb and the corresponding developed pupae on Day 17. Panel c, frontal picture of both left and right side of the uncapped brood comb after manual inspection.

Figure 1 shows the development from larval (Day 10) to pupal (Day 17) stage of the brood cells from five randomly selected sections of the comb. In total, despite the medical CT radiation dose applied on day 10, 105 out of 107 cells (98,1%) correctly molted into pupae as expected following the normal development pattern of honeybees^14^.

**Figure 2:**
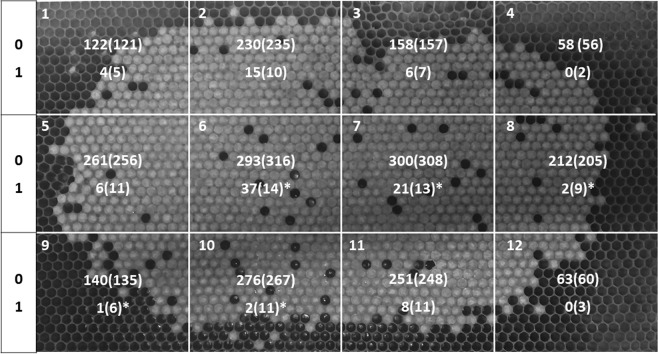
Brood area sections (1–12) and superimposed contingency table for absence (0) and presence (1) of observed and expected (in brackets) varroa mites. *Significant χ2values (P < 0.05).

A total of 2466 pupae were inspected for presence of varroa mite in their cells and the corresponding lengths were measured from the CT images. One-hundred two out of 2466 cells were infested by the mite, corresponding to a 4.1% total true brood infestation of the analyzed comb. Figure 2 summarizes the results from χ2 test by presenting the observed and expected frequencies of varroa mites in a contingency table. The association between presence and absence of varroa and the position of the cells in the twelve sections was statistically significant (χ^2^ = 75.41, DF = 11, P < 0.001). Moreover, considering the distribution of the presence of varroa within each section, the two central ones showed more varroa mites than expected (section 6: χ^2^ = 39.95, DF = 1, P < 0.001; section 7: χ^2^ = 4.49, DF = 1, P = 0.03). Besides, less mites than expected were observed in sections 8 (χ^2^ = 5.30, DF = 1, P = 0.02), 9 (χ^2^ = 4.04, DF = 1, P = 0.04), and 10 (χ^2^ = 7.85, DF = 1, P < 0.01).

The two central sections contained 651 cells whose 58 were parasitized resulting in a partial brood infestation of 8.9%. This value of brood infestation was higher compared to total brood infestation rate reported above (4.1%).

Results from each of the three statistical models showed that the stage of the pupae, the position in the brood area (i.e. the two central squares analyzed) as well as varroa mites had significant effects on the length of the pupae (P < 0.001). Each model showed that pupae at stage 8 were significantly shorter than pupae at stage 7. Statistically significant difference was also found between the length of pupae in square 6 and square 7. The pupae analyzed in square 6 were longer than pupae in square 7; this result could be explained by the fact that square 6 was facing the entrance of the hive, which was orientated to South and probably exposed to higher temperatures.

Table 1 reports the Least Square (LS) means of the length of the bee pupae estimated with each of the three models considering the variable varroa (V) in three separated categories: presence/absence of the mite, number of foundress mites and total number of mites found within the cell. LS means from the first model showed that the presence of varroa mite significantly affected the length of the pupal stage by a reduction of 0.35 mm (from 10.54 mm to 10.19 mm) which represents approx. the 3% of the average varroa mite free pupa length in our sample. In the second model the effect of varroa was considered as the number of foundress mites found in the analyzed cells. LS means for the length of pupae hosting one foundress mite was 10.20 mm and was significantly shorter compared to varroa free pupae (10.54 mm). The length of pupae parasitized by two or more foundress mites was 10.08 mm and significantly shorter than varroa-free pupae, but not significantly shorter than pupae with one foundress mite. Results from the third fitted model showed that the length of the pupae was significantly shortened also by the presence of more than three individuals within the same cell.

**Table 1:** LS Means (±SE) and relative number of observations (N) of length of pupa for the three categories of mite infestation: presence or absence; number of foundress mites; total number of mites.

Presence/absence model	N	LSMeans ± SE
0 – absence	593	10.54 ± 0.02^a^
1 – presence	58	10.19 ± 0.04^b^
**Number of foundress mites model**		
0 – absence	593	10.54 ± 0.02^a^
1 foundress mite	52	10.20 ± 0.04^b^
>=2 foundress mites	6	10.08 ± 0.11^b^
**Total number of mites model**		
0 – absence	593	10.54 ± 0.02^a^
1 mite	9	10.30 ± 0.09^ab^
2–3 mites	17	10.24 ± 0.07^b^
4 mites	18	10.16 ± 0.06^b^
≥5 mites	14	10.09 ± 0.07^b^

Means with different superscript are statistically different (P < 0.05).

## Discussion

The CT technology is increasingly used in scientific research about insects, and particularly the µCt scan and the 3D Phase-contrast X-ray computed tomography have been performed for anatomical studies and for the analysis of internal pathogens of honeybee individuals^12,15,16^. In this study, the length of developing pupae within intact brood using medical CT-scan technology was carried out. This would be a relevant new tool to allow morphological measurements of honey bee’s developing stages without uncapping the cells during *in vivo* studies. Pupa is the developmental stage of honeybee during which the insect is referred as quiescent and still. For this reason, we exclude that movement of the individuals are a potential source of artifacts in the CT images. Previous published observations carried out under laboratory conditions, confirmed that in the period of time between the prepupal ecdysis and the pupal ecdysis, the insects lay still on their back^17^. Moreover, the applied radiation dose did not seem to affect the normal development of brood from larval to pupal stage (Fig. 1c). The spatial distribution of *V. destructor* in the studied comb showed that varroa mites preferentially invaded cells in the inner brood area rather than infesting evenly the brood cells. This could suggest a preference of varroa mites for central brood areas, where temperatures are known to be kept slightly higher and more constant by worker honeybees compared to the periphery of the combs, even if different results are reported for varroa mites in tropical environment, where the development of the parasite seems to occur at a lower temperature compared to that in the brood^18,19^. The findings about higher infestation rate of the central sections of the comb also confirmed the importance of random sampling of manually inspected cells during brood mite monitoring.

Our results showed that the length of the pupae was influenced by the developmental stage, by the position within the brood comb area and by the parasite load. The length of pupae at stage 7 and stage 8 (post-capping days) was negatively affected by the presence of the mite, and became shorter the more mite individuals were present in a cell. Such an inverse relationship between the length of the pupa and the number of affecting mites could be linked to the nutritional behavior of the parasites on the developing honeybees. Indeed, varroa mites during their reproductive stage within the brood cell pierce the cuticle and feed on the developing honey bee^5^. Considering that the size of the pupae can be correlated with its weight, the above results agree with previous studies on the effect of parasitization on the weight of honeybees at their emergence^6,7,9^.

From the perspective point of view, our study suggests that CT-imaging could become a fast and non-destructive approach to explore the developing status of the honey bee brood stages. Medical CT-scan cost is clearly lower compared to micro-CT scan and has fallen significantly over the past few years. Besides, medical CT-scan application is increasing not only in clinical settings but also in animal production and industrial systems^13^. It is also worth remembering that unlike what happens in the current clinical practice, for honeybee colonies the medical CT scanner could host simultaneously up to 36 combs/scan, thus allowing the monitoring of several colonies by one scan.

## Methods

The experiment was carried out in June 2018 at the Faculty of Veterinary Medicine, University of Milano, Via dell’Università n. 6, Lodi, Italy. Pupae from one brood comb were analyzed. The brood comb belonged to a honeybee colony in good health status and headed by naturally mated queen. At the beginning of the experiment (Day 0), the queen was caged on an empty comb and released after 48 hours (Day 2). This procedure permitted to obtain a comb hosting eggs within a range of maximum two days’ age difference. The queen was caged in order to obtain the most coeval individuals within a comb to minimize any variation that could possibly arise from the presence of different developmental stages of the honey bee. Moreover, from a practical point of view, the choice was made to be able to foresee the age of developing insects under study. After queen release, the brood comb was put back into the colony to allow the further development of brood under natural condition. Then, the comb was subjected to two CT scans on Day 10 and Day 17, respectively. At the time of the second scan, a population of pupae aged between stages 7-days and 8-days after capping should be expected^20^. Before each scan, the comb was extracted from its colony and put into a polystyrene hive nucleus for immediate CT scan at the close Veterinary Faculty Hospital. The images were acquired with a 16-slices CT scanner (GE Brightspeed^®^, GE Healthcare Milano – Italy), using a high resolution filter. Scanning parameters were set as follows: kV = 120, mA = 250, slice thickness = 0.625 mm, pitch = 0.9375. During the scans, a collection of 1529 and 1452 images was acquired on Day 10 and on Day 17, respectively. After the first scan on Day 10, the comb was put back in the colony. On Day 17, the comb was subjected to the second CT scan and stored afterwards at −20 °C until manual inspection.

### Image analysis

The acquired CT scans were visualized with image viewer Weasis (version 2.0.5), a free software which permits to handle DICOM files (Digital Imaging and COmmunications in Medicine). The length of each pupa was assessed using the measuring tool provided by the software on a selected group of images. For the most accurate measurement as possible, successive images of the same individual were considered in order to carry out the measurement on the one showing the widest slice of tomographic volume of the pupa. Moreover, the exact coordinates of the measured pupa in the comb were extracted using Weasis (version 2.0.5) and the original position within the comb determined by tracing back the coordinates to a common reference point (i.e., the top left part of the comb). This permitted to classify the spatial position of every cell in an imaginary array considered during statistical analysis (Fig. 2).

### Comb manual inspection

In order to assess the developmental stage of the pupa and the infestation of varroa mite, each cell of the comb was individually and manually inspected. The wax cap of each cell was opened with a scalpel and the pupa was extracted using a pair of tweezers. The age of the pupae and the presence of varroa mites were recorded according to Büchler *et al*.^20^. In addition, when any mites were found, the number of foundress mites (i.e., adult females with offspring) and the number of progeny were recorded.

### Statistical analysis

For the analysis of the length of the developing honeybee pupae, different factors were considered. Firstly, the position of pupae within the brood was taken into account by sub-setting the brood area of both sides of the analyzed comb into 12 uniform squares by a grid containing 12 sections (3 rows by 4 columns, see Fig. 2). Secondly, the age of the pupae within each cell was considered as a variation factor. Lastly, the effect on the length of the pupae given by the presence of varroa in the cell was tested considering three different categories: i. Mite presence or absence; ii. Number of foundress mites; and iii. Total number of mite’s individuals found in the cell.

To test the relationship between the presence of *V. destructor* and the position of the cells within the 12 sections of the brood comb, a χ^2^ analysis was performed. This permitted to assess if varroa mite was distributed in a uniform way within the brood comb.

We chose to analyze the length of pupae situated in the two central sections of the comb assuming that such area shares a slightly higher and more constant temperature, which can influence the size of the developing insect^18,21–23^.

The following fixed model was fitted to data, using PROC GLM of SAS®^24^:

$${y}_{ijkl}=\mu +{S}_{i}+{A}_{j}+{V}_{k}+{e}_{ijkl}$$

where µ is the overall mean oh the length of the pupa, S refers to *i*^*th*^ section of brood in the comb (i = 1,2), A is the *j*^*th*^ developmental age of the pupae (j = 1,2), V is the *k*^*th*^ effect of varroa in the cell, and e is the random error term of the *l*^*th*^ observation (l = 1, 651).

As regards to the effect of varroa, firstly V was fitted as a binary factor indicating the presence or absence of varroa within the cell (k = 0,1). Secondly the number of foundress mites was considered, where V term varied between 0, 1 foundress mite and more than one founder (k = 1,3). Lastly, the effect of the total number of mites within the cell (foundress, son and daughters) was assessed considering V ranging from 0 to 5 individuals (k = 1, 6), where cells with 2 mites were pooled with cells with 1 mite and cells with more than 5 mites where pooled with cells with 5 individuals.

Least square (LS) means were separated by pair-wise t-test and Bonferroni adjustment was applied. Mean separation for main effects were performed on least square mean using PDIFF option of SAS^® ^^19^. Statistical differences were declared at P < 0.05.

## Data Availability

Raw data were generated at the Faculty of Veterinary Medicine, University of Milano. Derived data supporting the findings of this study are available from the corresponding author [E.F.] on request.
